# Safety for Recovery During Admission to Acute Mental Health Hospitals: A Constructivist Grounded Theory Study to Support Safety‐Focused Recovery‐Oriented Care in the Context of Risk Management Constraints

**DOI:** 10.1111/inm.70104

**Published:** 2025-07-31

**Authors:** Kris Deering, Chris Wagstaff, Richard G. Kyle, Ivor Bermingham, Jo Williams, Chris Pawson

**Affiliations:** ^1^ Nursing Academy: University of Exeter Exeter Devon UK; ^2^ Institute of Clinical Sciences, University of Birmingham Birmingham UK; ^3^ Service User and Carer Involvement Coordinator in the Southwest of England UK; ^4^ Centre for Psychosocial Research in Cancer, building 67/4055, School of Health Sciences, Faculty of Environmental and Life Sciences University of Southampton Southampton England UK; ^5^ Psychology Department University of the West of England Bristol UK

**Keywords:** acute mental health hospital, constructivist grounded theory, interpersonal therapeutic relationships, mental health recovery, risk management, sense‐making

## Abstract

Recovery during admission to acute mental health hospitals can involve supporting individuals in beginning to work towards a fulfilling life despite experiencing mental distress. This can help reduce risks such as suicide and aggression. However, restrictive risk management practices, such as physical restraint may increase during this period, and the distress caused by these practices can hinder a person's abilities to understand their recovery needs. Little is known about how recovery can occur safely within the context of such risk management restrictions. Therefore, this constructivist grounded theory study explored the perspectives of 15 individuals with hospital admission experiences about how they might begin their recovery journeys amidst these constraints. The study found that people could reconnect with their beliefs about a fulfilling life vicariously through interpersonal relationships. This facilitated a sense‐making process allowing individuals to better understand their recovery needs which were otherwise obscured by the complexity and intrusiveness of risk management practices. The theory comprised of four social processes: ‘treating me safely,’ when nurses began to understand those admitted as individuals; ‘outside world inside,’ which involved nurses helping the person to form meaningful connections to their personal world; ‘tangible hopefulness,’ where nurses raised the person's awareness of meaningful successes; and ‘scaffolding recovery,’ which built on the previous three processes, with the nurses helping individuals recognise the potential for working towards a fulfilling life. The study shows how supporting a relational approach to care may lessen people feeling unsafe when admitted to hospital, despite experiencing potentially distressing risk management practices.

## 1 Introduction

Recovery within acute mental health hospitals can focus on building a meaningful life and fostering hope for a fulfilling future, even when a person is experiencing severe mental distress (Murphy et al. 2023). Nevertheless, restrictive risk management practices during the admission period, such as observations and ward seclusion, can increase a sense of insecurity and feeling unsafe. These practices may lead to profound self‐doubt, hindering individuals' abilities to understand and address their recovery needs (Deering et al. 2024). Establishing therapeutic relationships not only enhances feelings of safety but also initiates the recovery process (Kendal et al. 2024). However, the dynamics of these interpersonal interactions, particularly during the admission period and within the constraints of risk management, remain underexplored. This study aims to theorise how nurses can develop interpersonal relationships that enable individuals to begin sense‐making their recovery needs despite constraining risk management practices. According to Battles et al. (2006), sense‐making involves abilities to find helpful meanings within events based on people's unique knowledge about how they understand their lives. To align with recovery‐based language, this paper will refer to those admitted to hospitals as ‘people’ ‘persons’ and ‘individuals,’ ensuring their identities are not reduced to a service or condition (Mental Health Coordinating Council 2022).

## 2 Background

This study explored how individuals may start to sense‐make their recovery needs upon admission to mental health hospitals, aiming to mitigate some of the negative effects of restrictive risk management practices. For over two decades, the recovery approach has been shaping international mental health services (World Health Organization [WHO] 2019). In stark contrast to traditional thinking around pathology which prioritises the absence of mental illness, recovery also focuses on enriching quality of life *despite* mental distress (Anthony 1993). Recovery represents a shift from an overemphasis on the medical model, which prioritises medicines to address mental illness and risks, towards a more relational, person‐centred approach (Winsper et al. 2020). Distinctions can be made about how recovery is viewed with'clinical recovery’ led by clinicians, while'personal recovery’ is led by individuals experiencing mental distress (Slade 2009). However, these approaches can converge, and through collaboration, knowledge can be shared about what might help individuals under mental health services to have a voice around their own care needs (Dubreucq et al. 2022). Consequently, practices informed by both recovery approaches, termed recovery‐oriented care, may begin to improve quality of life even when experiencing severe mental distress (Murphy et al. 2023).

Despite the recommendation to promote recovery from the first day of admission (WHO 2019), tensions can exist between clinical approaches to mitigate risks and enabling people to commence their recovery journeys. These tensions are particularly evident during the admission period to a mental health hospital in which risk management may increase in response to clinical concerns about the risks associated with mental illness (Slemon et al. 2017). Restrictive practices to address risks within hospitals (including suicide, self‐harm, and violence) can also often be prioritised over recovery‐oriented care approaches (Johnson et al. 2022). Excluding individuals from their own risk assessment and risk management plans may stem from clinical concerns that conflicts over discrepant views of risk could emerge and escalate during this process, increasing risks such as aggression (Kim and Nam 2024). This exclusion not only hampers the individual's voice, which is essential for informing recovery‐oriented care, but also perpetuates perceptions of oppressive risk management practices among those in hospital (Deering et al. 2024). These include observations, ward seclusion, physical restraint, and forced medication (Slemon et al. 2017).

The concept of safety can also be a contentious issue in mental health hospitals. Clinicians may believe that individuals recently admitted are too disoriented by their mental illness to understand their own safety needs (Kendal et al. 2024). However, safety can be more than clinical risk concerns about deliberate harm to self and others (Deering et al. 2019). Adopting a recovery‐oriented stance suggests that psychological safety must also be considered, providing opportunities for individuals to discuss their personal needs without jeopardising their interpersonal world (Hunt et al. 2021). Through this recovery‐oriented lens, individuals receiving hospital care should feel safe to begin working towards a meaningful life—and this, too, is seen as a safety concern expressed by those admitted to hospital (Thibaut et al. 2019).

## 3 Aim

The research team has previously reported that individuals recently admitted to hospital often find risk management practices to be obscure, because the reasons for these practices are not explained. Risk management practices such as ward seclusion also felt insensitive to personal needs; consequently, individuals experienced insecurities about their situation, which hindered their abilities to understand and address their recovery needs (Deering et al. 2024). This paper builds on these findings and addresses some constraints of risk management by examining participants' experiences of interpersonal relationships and their potential role in sense‐making recovery during hospital admissions. These experiences were drawn from adult acute mental health hospitals in the southwest of the United Kingdom (UK).

## 4 Methods

**FIGURE 1:**
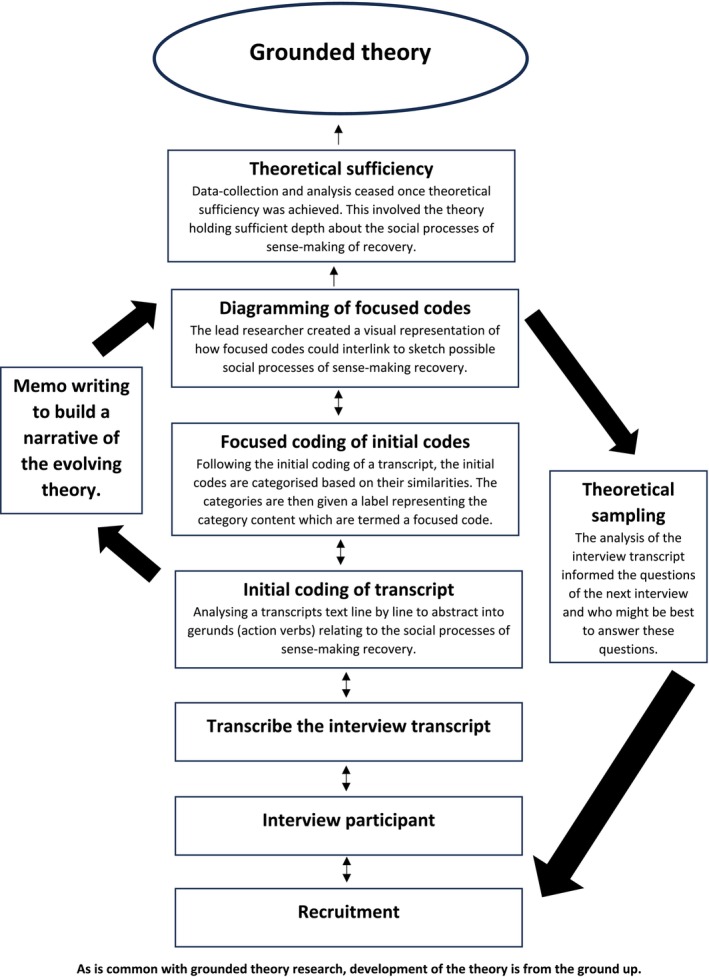
Outline of conducting the study. As is common with grounded theory research, development of the theory is from the ground up.

Given that interpersonal relations are crucial for recovery and a key component of mental health nursing (Llewellyn‐Beardsley et al. 2019), the research adopted a constructivist grounded theory methodology. This methodology aims to elicit new understandings of social processes, informed by how individuals interact and establish relationships (Charmaz 2014). This approach aligns with the study's aim to understand how interpersonal interactions may facilitate the sense‐making of recovery. As there is a lack of research about recovery in the context of risk management constraints, particularly during the hospital admission period, the methodology was also selected to generate a theory to inform future research and mental health care. The study adopted the position that recovery is experienced by people experiencing mental distress, and it is therefore their perspectives that are important to understand. Despite risk management significantly impacting the lives of those receiving hospital care, research in the field often focuses on mental health practitioners' views (Coffey et al. 2017). Hence, awareness of this knowledge gap further justified seeking only the perspectives of people with lived experiences of admissions to mental health hospitals (Figure 1).

### 4.1 Participants

Recovery can be seen as a developmental journey. To incorporate different views informed by this progression, a sampling strategy was devised to include people at various stages of their recovery. Notably, individuals recently discharged from hospital and individuals who were fully discharged from statutory services and receiving support from a community support group. Arguably, members of the community support group may have a cultivated understanding of their recovery needs compared to those recently discharged from hospital (Llewellyn‐Beardsley et al. 2019). Other inclusion criteria specified that participants were not experiencing debilitating mental distress and could make informed decisions (see Table 1).

**TABLE 1:** Inclusion and exclusion criteria for sample.

Inclusion criteria	Exclusion criteria
Has experiences of acute mental health difficulties	Not discharged from an acute adult mental health hospital [to not add a burden]
Experienced at least one admission to an adult mental health hospital	Experiencing debilitating acute mental health illness [to not add burden]
People from the community support group required to be fully discharged from mental health services	Advised by clinician(s) or the community support lead that the person is unable to participate
People aged 18 and above to fit with the criterion of accessing adult acute mental health hospitals within the United Kingdom	Has another condition affecting mental capacity

Theoretical sampling was employed with the analysis of interview transcripts guiding the subsequent data collection. Participants were asked questions based on the ongoing analysis of the theory, and the most appropriate participants were identified to answer these emergent questions to enhance theory development (McCrae and Purssell 2016). Due to ethical approval restrictions, the study could not directly recruit or advertise to potential participants in case they were too unwell to participate. To address this, the community mental health team and the community support group served as Participant Identification Centres (PICs) (National Health Service Health Research Authority 2024). Nurses and the support group lead at the PICs shared the study information sheet with individuals who met the inclusion/exclusion criteria. Thereafter, the principal researcher (lead author) was informed of those interested in participating. To minimise coercion, the principal researcher conducted phone calls with potential participants before obtaining consent to gauge their interest in the research (Xu et al. 2020). As a result, two individuals chose not to participate in the study for undisclosed reasons. A group of lived experience experts also reviewed and approved the theoretical sampling method and initial questions commencing the study (shown below) as part of the ethical approval process. This was to ensure that the interviewing and recruitment approach would not be detrimental to participants.

In the results section, participant pseudonyms and chronological number of their interview will be highlighted as shown in the table (P = participant, Number = order of interview). This is to help track how quotations from the interview transcripts informed the data analysis.

### 4.2 Interviews

Fifteen people were interviewed in person between July 2020 and November 2022 using a digital recorder (see Table 2). Interviews lasted between 40 and 143 min (averaging 80 min), resulting in over 28 h of recorded data. The first five interviews started similarly with the questions: “How do you understand your recovery?”, “What is risk management in hospital?”, “How did risk management influence your recovery?”, and “How did engaging your recovery occur?”. Theoretical sampling then shaped further interview questions for the remaining participants based on the data analysis of the previous interviews. The final two participants were also asked to share if they had any critique about the findings to improve the theory's authenticity according to their recommendations.

**TABLE 2:** Characteristics of participants.

Averages/percentages, interview number	Pseudonyms (names agreed with the participants)	Self‐identified gender	Age Ranges	Self‐identified ethnicity	Self‐identified mental health condition	Year of most recent acute mental health hospital admission	Approximate length of stay in an acute mental health hospital
Averages and percentages		Male: 73% Female: 27%.	Average age: 38	White British: 80% White Scottish: 6.6% White South‐African: 6.6% Black Eastern African: 6.6%	Psychosis: 73% Bipolar: 6.6% Borderline personality disorder: 6.6% Post traumatic Stress Disorder: 6.6% Disagreed with diagnosis: 6.6%	Average year of admission to an acute mental health hospital: 2016	Average length of stay in an acute mental health hospital: 7.5 months
Interview 1:	Dave	Male	20–29	White British	Psychosis	2016	5 Months
Interview 2:	Peter	Male	50–59	White South African	Psychosis	2017	9 Months
Interview 3:	Jack	Male	50–59	White British	Bipolar	2016	12 Months
Interview 4:	George	Male	20–29	White British	Psychosis	2014	14 Months
Interview 5:	Mary	Female	50–59	White Scottish	Borderline Personality Disorder	2010	3 Months
Interview 6:	Richard	Male	60–69	White British	Post‐Traumatic Stress Disorder	2005	6 Months
Interview 7:	Sam	Male	20–29	White British	Psychosis	2019	5 Months
Interview 8:	Aisha	Female	30–39	Black Eastern African	Psychosis	2019	7 Months
Interview 9:	Sarah	Female	40–49	White British	Psychosis	2019	8 Months
Interview 10:	Kate	Female	30–39	White British	Psychosis	2020	8 Months
Interview 11:	Jim	Male	50–59	White British	Believed it a spiritual conflict though stated staff called it schizophrenia	2019	12 Months
Interview 12:	Tom	Male	30–39	White British	Psychosis	2020	11 Months
Interview 13:	Roger	Male	20–29	White British	Psychosis	2018	4 Months
Interview 14:	Derek	Male	20–29	White British	Psychosis	2018	Fourth Months
Interview 15:	Joe	Male	20–29	White British	Psychosis	2018	5 Months

### 4.3 Data Analysis

Each interview transcript was analysed before the next interview took place to support theoretical sampling, beginning with initial coding followed by focused coding. Initial coding involved examining transcript text line by line and abstracting insights about recovery into words or short sentences. The aim was to generate gerunds (action verbs), as these verbs can guide the shaping of social processes (Apramian et al. 2017). Initial codes were then categorised based on their similarities to create focused codes, which are abbreviated labels signifying a category's content (Charmaz 2014). Subsequently, focused codes were diagrammed by sketching visual representations of how these codes might interlink, connected by symbols such as arrows. These diagrams built upon those from previously analysed transcripts. As the theory evolved, these linkages gradually developed an understanding of the social processes involved in sense‐making recovery through interpersonal relationships.

**FIGURE 2:**
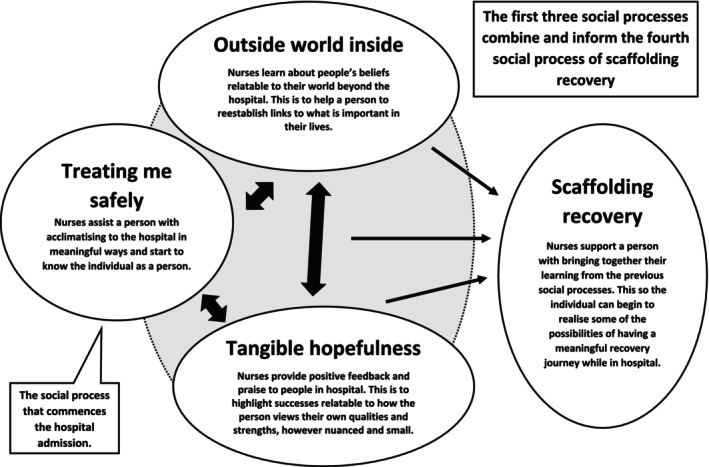
Overview of the theory.

Memos were also written to explain how focused codes interlinked, developing a narrative that elucidates the theory's properties (with the finalised version in the results section). Constant comparison aided this process, involving systematically analysing data by continuously comparing data to data to generate the theory. For example, focused codes, diagrams, and memos from different interview transcripts were frequently compared to construct a coherent narrative as the theory developed (Birks and Mills 2023). Data collection and analysis ceased once theoretical sufficiency was achieved. This meant the theory had sufficient depth to understand how the social processes within interpersonal interactions could aid the sense‐making of recovery (Figure 2 and Table 3).

**TABLE 3:** Example of initial coding and a focused code in the study.

Interview	Transcript text	Initial Codes	Focused code (the label given to the categorising of the initial codes in the previous column)
**Interview 1: Dave**	“I have been so encouraged by, I**r, and mmmm N*****a from Sxxxxxxxxd, they are great photographers, but overtime that has changed […]*”*	**Sharing hobbies with staff**	
**Interview 2: Peter**	*“I said wouldn't it be nice just get nice and clean, go and run yourself a bath some…some smellies and salt and things* […] *she came back every evening at night and bring me sandwiches* […] *really really nice* […]*”*	**Civility (activities helping others)**	**Meaningful activities**
**Interview 3: Jack**	*“I think there was a suggestion that there might be a dialogue of somebody um like service users being involved with actually talking about risk”*	**Actively listen to patient voice**	
**Interview 4: George**	*“Just even if it is a nice board game or something that get people sat down, gets people together that wouldn't normal talk they sit down to play a game of cards yeah”*	**Sparking interaction**	

## 5 Results

### 5.1 Overview of the Theory

Participants shared that when interpersonal relationships vicariously reconnected them to personal beliefs about having a fulfilling life (relating to their lives outside the hospital), it helped the sense‐making of their recovery needs. This sense‐making process involved understanding how these beliefs could begin to inform a meaningful recovery during the hospital admission period. When beginning to sense‐make recovery, it also appeared to alleviate feelings of insecurity caused by restrictive risk management practices. Hence, by starting such a sense‐making process, participants suggested that they began to feel safer about their experiences in the mental health hospital. An iterative process also seemed to occur in which feeling safer further enabled abilities to understand personal recovery needs.

### 5.2 Social Process 1: Treating me Safely

‘Treating me safely’ was identified as the first theoretical social process. People often felt unsafe upon admission, partly due to the anxieties and insecurities seemingly caused by restrictive risk management practices. These practices appeared alien compared to the person's world outside the hospital ward. Therefore, the social process of ‘treating me safely’ served to help individuals feel safer by assisting them in acclimating to the hospital environment. This was experienced by participants when nurses shaped a mutual understanding. Notably, ‘Jim’ (P11) suggested that his admission would have been better if he had been supported in practicing his faith as a way to acclimate to the hospital. Such support might have made the admission resonate more with Jim's needs outside the hospital, while also adding meaningful purpose to his hospital stay.

It's [me] getting used [via support] to practising my faith […]. It gives you purpose about the admission. P11

Exploring a person's beliefs, even if clinically perceived to be influenced by mental illness, could serve as a benchmark to enhance the compatibility of communication, ensuring the individual's understanding. Individuals realised that when nurses attempted to understand their beliefs, they were trying to know the person beyond a series of psychiatric conditions. It was also part of beginning to shape a mutual and evolving understanding between nurses and patients, which seemed vital to forming some stable ground of communication in which ideas about recovery needs could start to form.

Feeling safe. Just taking that time to get to know you, and not always in an abrupt way. P9

Cultivating control was an integral part of ‘treating me safely.’ Participants spoke about feeling safer when able to relinquish some control into the hands of the nurses when first admitted. It was also valuable for nurses to provide individuals with opportunities to express their views on experiencing mental distress. According to Aisha (P8), such opportunities could lead to meaningful conversations about recovery needs.

I need to let everything go and you calm down, that's all. The messenger just wants to deliver the message [about mental distress] and [staff] need to listen to what I have to say, […] then we can talk about […] recovery. P8

Understanding the rules governing the hospital helped individuals gain a sense of control by allowing them to grasp expectations, even if they disagreed with the mental health nurses. This knowledge began to create a common ground between nurses and individuals, facilitating a mutual understanding of what was feasible at that time and how it could support recovery. For example, when nurses allowed interactions within time limits set by the individual, it helped people feel more in control and begin exploring personal qualities that could inform their recovery needs. When these qualities resonated with the person's life, it initiated a trust‐building process, making individuals feel safer about starting their recovery. Even when unsuccessful, nurses' attempts to explore an individual's thoughts about their recovery needs contributed to building trust and feeling safer. This was because there was intrinsic value in seeing mental health nurses trying to use the person's knowledge to find ways to help.

Build the trust [through] open conversations […] where they feel comfortable […] and acknowledged. P1

Important to start getting what [the hospital admission] is all about, why I am there, what do you [nurses] do. P14

### 5.3 Social Process 2: The Outside World Inside

The ‘outside world inside’ fostered a connection to the individual's world beyond the hospital. The person's beliefs were further explored through interpersonal interactions to identify what might be fulfilling and aid understanding of their recovery needs. The hospital seemed isolated from an individual's world, not only due to the inability to leave but also because hospital practices lacked personal relevance to people's lives. This was particularly salient when encountering risk management practices that limited the liberties they were accustomed to outside the hospital. By connecting to a person's beliefs, activities could be devised that developed meaningfulness for the individual. It was not necessary to replicate all activities important to a person as some may be hazardous within a hospital setting. However, nuances of these activities could still be utilised to vicariously aid the person to reconnect to beliefs about having a fulfilling life. For example, Roger (P13) desired activities that he could personally relate to, for it could help with sense‐making his recovery needs. Participants shared that when nurses employed such a process, it also enhanced their motivation and feelings of safety, especially when the activities felt more familiar to their lives.

[Recovery] was around my life you know […] I have got such diverse things that I love doing yet I can do [linking to these pastimes] are important […]. P13

By noticing details such as people wearing sports jerseys or watching specific television shows, nurses could gain insights into what was conventional and important in their world. These observations could then become topics for inquiry about beliefs that might aid in a person's recovery. For example, nurses could attune to the subtextual meanings voiced by individuals like ‘Jack’ (P3), who figuratively referred to football as “real life.” This suggested that the sport held significant personal value for him and, with further inquiry, could provide insights into some of Jack's recovery needs.

I then said […] sorry to bring football into it and it's not a game it's real‐life. P3

Clues could also emerge about a person's experiences of mental distress. For example, ‘Aisha’ (P8) expressed the burden she felt from Jesus commanding her to share the word of God, which nurses struggled to relate to. According to Aisha, nurses perceived her experiences as a risk due to psychosis, and forced medication seemed to be the primary line of treatment. However, Aisha said a nurse had also invited a chaplain to speak to her and by already inhabiting a similar world, the chaplain seemed less concerned about Aisha's experiences being ‘hallucinations.’ Through the chaplain already having a developed relatedness to Aisha's religious views, this allowed for discussions to be more in touch with her religious beliefs at the time. It seemed that the approach enabled Aisha to consider her recovery in broader terms, given that she reconnected to beliefs she had before the admission, in which Aisha thought Jesus was compassionate and would not necessarily be placing such a burden on her.

The chaplain came to my level […] in how you can understand […] he took me as a whole me and hearing Jesus, which to me was real at the time. P8

### 5.4 Social Process 3: Tangible Hopefulness

‘Tangible hopefulness’ involved further fostering a connection to a person's world beyond the hospital. This social process emphasised meaningful yet small‐scale successes that were realistic and addressed some of the insecurities arising from risk management, while also raising hopefulness about the future. By learning and developing awareness of personal recovery needs, hope became tangible. This was due to nursing interactions starting to seem more authentic to the person, helping to begin conversations to sketch out subtle aspects of what their recovery might entail. These could be consolidated via praise when the nursing team spotted the individual doing something that the person considered important. Hence, the social process could bolster the patient's confidence that despite the possible despondency of the admission, progression could still be part of their reality.

You just sort of chat about your successes […] to see hope. P2

Little things keep you motivated and going, and makes you feel valued and safe so if you have that recognition you know to progress along this journey, you will get stronger. P6

An example was ‘Sam’ (P7), who wanted to help people in hospital by sharing his biscuits. He realised this was an achievement when he received praise for his actions. It seemingly related authentically to Sam, for he wished to help others which played into his beliefs about having a fulfilling life. Hence, to applaud this achievement suggested a relatedness to Sam personally as it involved an attribute deeply important to him. Moreover, the praise highlighted that his integral sense of self was not lost owing to mental illness.

It's sort of helpful to me to see me being human […] if you want a biscuit have a biscuit […] staff […] thanked me […] it felt good. P7

‘Tangible hopefulness’ involved nurses providing praise for unplanned feats, as it indicated to a person's volition with doing an activity that they found meaningful in that moment. By drawing attention to actions via praise when individuals were engaged in activities seen to be important to them, it helped the person connect the praise to their actions. It reminded that authentic choices could still be made about how to meaningfully live despite mental distress and could consolidate what the person perceived as their strengths. Motivation could also further increase, for intrinsic value existed through how the praise aligned to actions which could develop a quality of life, adding a purposefulness to the care provided. However, when the nurses' focus was limited to mitigating risks, it could lead to a waning motivation, particularly through the use of restrictive risk management practices. This was because such practices had little association to a quality of life as accustomed to outside of the hospital.

Helped to realise that little things were possible for I discovered I liked it already and this kept me going. P9

### 5.5 Social Process 4: Scaffolding Recovery

‘Scaffolding recovery’ involved individuals beginning to identify elements important in their lives and piecing these together to foster a growing hopefulness about achieving fulfilment. To reach ‘scaffolding recovery,’ it was necessary to first start developing a sense of safety and hope through the previous social processes, enabling individuals to begin sense‐making their recovery needs. This sense of safety and hope helped address feelings of despondency, particularly considering the constraints of risk management practices. Participants also suggested that their insecurities could start to diminish, as reaching ‘scaffolding recovery’ implied that they were beginning to believe that the hospital might have some usefulness.

You are restoring…what they are doing is trying to restore the files back…into your brain […] you can make sense of the [hospital] situation. P3

Like talking about what helps and making that a recovery plan like “how was that?,” “how did that go?” P15

Motivation could additionally increase as a picture materialised about the possibilities of recovery relatable to the person's life beyond the hospital. ‘Mary’ (P5) and ‘Kate’ (P10) proposed that progress was difficult to fully remember due to their insecurities, partly caused by risk management practices. As Mary suggested below, documenting progress was an essential part of ‘scaffolding recovery.’ For example, writing, drawing, or painting pictures about an individual's progressions (informed by personal views) helped people to remember their successes. By doing so, an individual was aided to visualise the possibilities of working towards a fulfilling life while in hospital, despite risk management restrictions. It was also seen as an opportune time to inform risk assessments, supported by how the social processes to reach ‘scaffolding recovery’ enabled a person to feel safer about disclosing more personal safety concerns. ‘Richard’ (P6) suggested that this point could be an opportune time to incorporate meaningful activities into risk assessments, given the enhanced ability for people to sense‐make their recovery needs. This approach could not only reinforce important beliefs about having reasons to live, thereby reducing risks such as suicide, but also mitigate potential aggression stemming from frustrations about being in the hospital.

But if you write it down, you are going to remember it […]. I learnt about my [recovery] needs […]. P5

When doing the risk assessment, the question not asked, is what [are] your hobbies and interests before coming onto the ward, we are reintroducing them to it, and that is where they are getting the positivity from it […] also lessens barriers. P6

[…] A big help [recovery] just clicks […] and helps to make it fit. P10

Engaging patients [can help] feeling less frustrated and lashing out. P14

Through the consolidation of ‘scaffolding recovery,’ participants expressed that their beliefs were not only strengthened to inform the sense‐making of recovery, but they could also begin to regain a sense of self in what was meaningful. As Tom (P12) indicated, individuals could begin to reconnect with their personal identities beyond the limitations of mental illness. However, if sense‐making recovery is not supported, Tom suggested that individuals might feel lost and directionless in establishing a meaningful recovery journey while in the hospital.

More about keeping in touch with your identity as who you are, this is who I am, this is what I do […]. If you don't know where you're going, you're lost. P12

## 6 Discussion

This study generated a grounded theory helping to understand what might assist individuals in starting their recovery during the hospital admission period, even within the context of risk management constraints. Nurses developing relationships which vicariously connected people to their beliefs about a meaningful life supported a sense‐making process about the person's recovery needs. Four social processes underpinning the theory facilitated a learning process where individuals began to recognise what was important in their lives, while also counteracting some of the insecurities caused by restrictive risk management practices.

Interpersonal relationships are the cornerstone of mental health nursing and play a crucial role in promoting a person's recovery. However, this study is original as the way in which interpersonal relationships are enacted to aid recovery, particularly during the hospital admission period, has been under‐theorised and underreported (Waldemar et al. 2018). The study demonstrated that individuals could begin their recovery despite experiencing constraining risk management practices upon admission. However, aiding a person in sense‐making their recovery needs appears to be a missing step when reviewing the literature on recovery‐oriented care within hospitals. This gap can partly be attributed to paternalistic care practices, where nurses may devise recovery care plans before a person is even admitted, and these plans do not always focus on personal needs (Raphael et al. 2021).

Despite the above, the process of sense‐making in what aids a person's recovery shares similarities with other research, although the studies do not specifically relate to hospital care. Hence, it adds further uniqueness to our study. The Power Threat Meaning Framework (Johnstone and Boyle 2018) and Experience Recognition (Iversen 2018) both propose that interpersonal relations are vital for sense‐making to address problematic situations for people experiencing mental distress. Whereas Experience Recognition helps to frame people's experiences in ways to aid their sense‐making about personal difficulties (Iversen 2021), the Power Threat Meaning Framework suggests that sense‐making is developed via recognition of an individual's beliefs (Johnstone and Boyle 2018). Thus, similar to the findings of this study, this Framework highlights that understanding the interpretations of mental distress is vital (negative power), alongside building a personally coherent story of hope around strengths defined by the individual (positive power) (Boyle 2022). Indeed, neglecting the importance of this interplay at the heart of the connection to what a person defines as meaningful may, as Castelli Dransart (2013) further suggests, increase serious risks such as suicide.

This study has demonstrated that alternative actions can be provided to support recovery‐oriented care, even when a person is encountering risk management restrictions. In this context, the seminal literature on Peplau's (1952/1992) ‘theory of interpersonal relations’ and Aponte's (1982) ‘therapeutic use of self’ remains relevant. Nurses must be mindful of the significance of therapeutic relationships in care practices. Furthermore, the therapeutic use of self indicates to having self‐awareness about how nurses' duties to mitigate risks, may limit abilities to assist a person's recovery. As the theory showed, this can be addressed by involving the community around the hospital and bringing in individuals (such as a chaplain) who can better relate to a person's beliefs about what may personally help (Johnson et al. 2022). However, for a person to start a meaningful recovery journey from the first day of hospital admission, nurses may need to reconsider how they mitigate risks. Failure to challenge the restrictiveness of risk management can result in negative consequences, including potentially depriving a person of care that is personally meaningful to their life (Bennetts et al. 2024).

### 6.1 Limitations

The sample lacked diversity in terms of ethnicity, which was partly influenced during the study period by the COVID‐19 pandemic. It was noted early in the study that, despite efforts to improve diversity, members of more diverse groups were unable to participate due to issues associated with the virus. This is supported by international research indicating that people from ethnic minority groups were disproportionately affected by the COVID‐19 pandemic (Tai et al. 2022). Additionally, the recollections of hospital admissions may have changed over time, impacting the veracity of the findings. Another limitation was that the participants were recruited from one geographical region. Although recruitment was from the only community mental health team in the area, this suggests the study lacked generalisability. For instance, the theory may not reflect the views of all individuals recently admitted to inpatient settings.

## 7 Conclusion

Commencing a recovery journey upon recent admission to an acute mental health hospital can be challenging. This difficulty arises not only from experiencing mental distress but also from the constraints imposed by risk management practices, which can hinder abilities to make sense of a valuable recovery journey. Through a grounded theory methodology, this study cultivated a rich understanding of how interpersonal relationships initiated the sense‐making of recovery needs by vicariously connecting individuals to their beliefs about a meaningful life. As a result, this counteracted some of the constraints imposed by restrictive risk management practices. However, given that nursing work often focuses on mitigating risks within mental health hospitals, it is crucial to review and limit restrictive risk management practices. Instead, there ought to be more focus, whenever possible, on employing interpersonal approaches that can better support individuals in their journeys towards a meaningful recovery.

### 7.1 Relevancy to Clinical Practice

Tensions exist in mental health hospitals between restrictions to limit clinical risk concerns against recovery‐based relational approaches. However, interpersonal relations are vital as part of engaging recovery. This study explained the social processes that mental health nurses might adopt to reach a position where, through therapeutic relationships, people are more able to begin understanding their recovery needs. Initiating recovery‐oriented care appears to firstly involve a sense‐making process to reconnect with beliefs about a fulfilling life to feel safer about the admission. Hence, when relationships connect to meaningful beliefs, there is potential for individuals to begin understanding the possibilities of a valuable recovery journey that enables them to make sense of how the hospital admission could be personally helpful.

## Author Contributions

K.D. conducted the study, C.W. and R.G.K. contributed to authoring the paper, I.B. reviewed the analysis while C.P. and J.W. edited and advised on the paper as PhD supervisors.

## Ethics Statement

The study received ethical approval from the UK Health Research Authority (Research Ethics Service Reference: 19/SW/0174. Date 12/11/2019).

## Consent

Informed consent was utilised to ensure participants understood the purpose the study while notified that they could leave the study at any time without consequence. Debriefs were available if the participants encountered any distress during the interviews. Participants were also offered to check the accuracy of their interview transcripts and gave consent to disseminate the study findings for publication.

## Conflicts of Interest

The authors declare no conflicts of interest.

## Data Availability

The data that support the findings of this study are available from the corresponding author upon reasonable request.
